# Effect of a hearing loss care education program for older adults targeted at visiting nurses: A cluster randomized controlled trial

**DOI:** 10.1371/journal.pone.0339478

**Published:** 2026-01-27

**Authors:** Sumiyo Nabeshima, Saiko Sugiura, Kiyomi Yamada, Sayuri Sable-Morita, Yukako Ando

**Affiliations:** 1 Faculty of Nursing, Kinjo Gakuin University, Nagoya, Japan; 2 Department of Epidemiology of Aging, Research Institute, National Center for Geriatrics and Gerontology, Obu, Japan; 3 Department of Otorhinolaryngology, National Center for Geriatrics and Gerontology, Obu, Japan; 4 Kariya Hearing Clinic, Kariya, Japan; 5 Department of Nursing, Seirei Christopher University, Hamamatsu, Japan; 6 Department of Nursing, Chubu University, Kasugai, Japan; 7 Doctoral Program, Nagoya City University Graduate School of Nursing, Nagoya, Japan; Teikyo University Hospital Mizonokuchi, JAPAN

## Abstract

Hearing loss in older adults is a risk factor for depression, cognitive decline, frailty, and other conditions, and it has various negative physical and psychological effects. Hearing loss care requires the support of medical professionals; however, most hearing care education is targeted at professionals working in hospitals and care facilities, with few interventions aimed at visiting nurses. To address this gap, we developed the “Hearing Loss Care Education Program for the Older Adults” to improve visiting nurses’ skills in managing hearing loss. This study aimed to evaluate the effectiveness of the program. This cluster randomized controlled trial was registered in the UMIN Clinical Trials Registry (UMIN000053337) and conducted across 22 visiting nursing stations: 11 in the intervention group (60 visiting nurses) and 11 in the control group (42 visiting nurses). The intervention consisted of on-demand lectures and technical exercises, followed by a 3-month follow-up period. The primary outcome was the “implementation of hearing care,” whereas the secondary outcomes were “knowledge of hearing care” and “confidence in implementing hearing care.” Between-group differences were analyzed using generalized estimating equations. As part of the primary outcome, a significant increase in the implementation of “hearing screening” was observed in the intervention group (odds ratio 8.35, 95% confidence interval 3.60–19.34, p < 0.001). Additionally, the intervention group demonstrated significantly higher “knowledge of hearing care” and “confidence in implementing hearing care.” These findings suggest that the program effectively enhanced visiting nurses’ knowledge and confidence in hearing care and supports the integration of hearing screening into their routine practice.

## Introduction

The prevalence of moderate-to-severe hearing loss increases with age, with a global prevalence of approximately 400 million [1]. In Japan, the prevalence of age-related hearing loss in older adults in their 70s and 80s is approximately 30% and 60%, respectively [2]. Moreover, with the aging of the Japanese population, an increase in the number of older adults with hearing loss is expected. Hearing loss is also a risk factor for depression [3] and cognitive decline [4]. There have been reports of a relationship between hearing loss and social isolation, including reduced participation in social activities and increased feelings of loneliness [1]. Older adults with hearing loss have a higher incidence of new cases requiring care than those without hearing loss [5]. Therefore, addressing hearing loss is important for maintaining independent living in older adults. Intervention studies on hearing care for nurses have been reported previously; however, these studies have focused on specific care skills, such as external auditory canal care [6] and communication [7]. Furthermore, educational interventions in hearing care have not yet been implemented for healthcare professionals in Japan.

The results of our previous study examining the current state of hearing loss care provided by visiting nurses to older adults indicated that the knowledge and implementation rates of hearing loss care among visiting nurses was approximately 50% [8]. Visiting nurses reportedly face difficulties with hearing aids and ear cleaning [9]. Therefore, we developed a “Hearing Loss Care Education Program for the Older adults” program targeting visiting nurses. This study aimed to clarify the effectiveness of this program. Participation in this program was expected to enable visiting nurses to acquire appropriate hearing loss care skills, thereby contributing to improvements in home nursing care.

## Methods

### Study design

This study was designed as a cluster randomized controlled trial involving two groups of visiting nursing station units. The findings are described in accordance with the Consolidated Standards of Reporting Trials (CONSORT) guidelines outlined in S1 File.

### Ethical approval

This study was approved by the Graduate School of Nursing, Nagoya City University Research Ethics Review Committee in November, 2023 (23025). This study was registered with the UMIN Clinical Trials Registry in January 2024 (ID:UMIN000053337/URL: https://center6.umin.ac.jp/cgi-open-bin/ctr/index.cgi). The purpose and methods of this study, along with the confidentiality of personal information, were explained orally and in writing to the administrators of each facility, and permission was obtained. Visiting nurses were provided with the same explanation, and written consent was obtained from all participants. Participants were informed that they could withdraw their consent at any time, both verbally and in writing. Consent was obtained by checking the consent confirmation box on the screen before starting the web survey. At the end of the study, the individuals in the control group were offered an opportunity to participate in the intervention.

This study was conducted in accordance with the principles of the Helsinki Declaration.

### Participants

Visiting nursing stations were defined as clusters, with each facility comprising approximately five visiting nurses. The intra-cluster correlation was set at 0.1 based on similar studies. The effect size was set at 0.5, indicating a moderate effect [10]. Assuming a significance level (P-value) of 0.05 and a statistical power of 0.8, the required number of clusters per group was calculated to be approximately 8.79 [11]. Therefore, nine facilities per group, i.e., 18 facilities in total (90 home-visit nurses), were deemed necessary. Facilities within Aichi Prefecture were recruited using snowball sampling. Stations specializing in pediatric or mental healthcare were excluded because they do not routinely provide visiting nursing care to older adults. To account for an anticipated dropout rate of approximately 30%, we aimed to recruit 65 participants per group. Accordingly, we targeted 13 facilities per group, totaling 26 facilities (130 visiting nurses), and solicited applications from these facilities.

### Randomization

Visiting nursing stations were the units of randomization to minimize the potential risk of contamination between programs and imbalance between the study arms. The 26 visiting nursing stations that obtained permission were stratified into three groups based on the number of affiliated visiting nurses: 12 facilities with two to four nurses, 10 facilities with five to eight nurses, and four facilities with nine to 11 nurses. One person who was not involved in the study used a die to randomly assign 13 facilities each to the intervention group and non-intervention groups (odd numbers: intervention group; even numbers: control group; Block randomization was performed within each stratum to equalize the number of facilities in each group). The results of randomization were communicated to visiting nurses through facility administrators.

### Intervention

**Fig 1:**
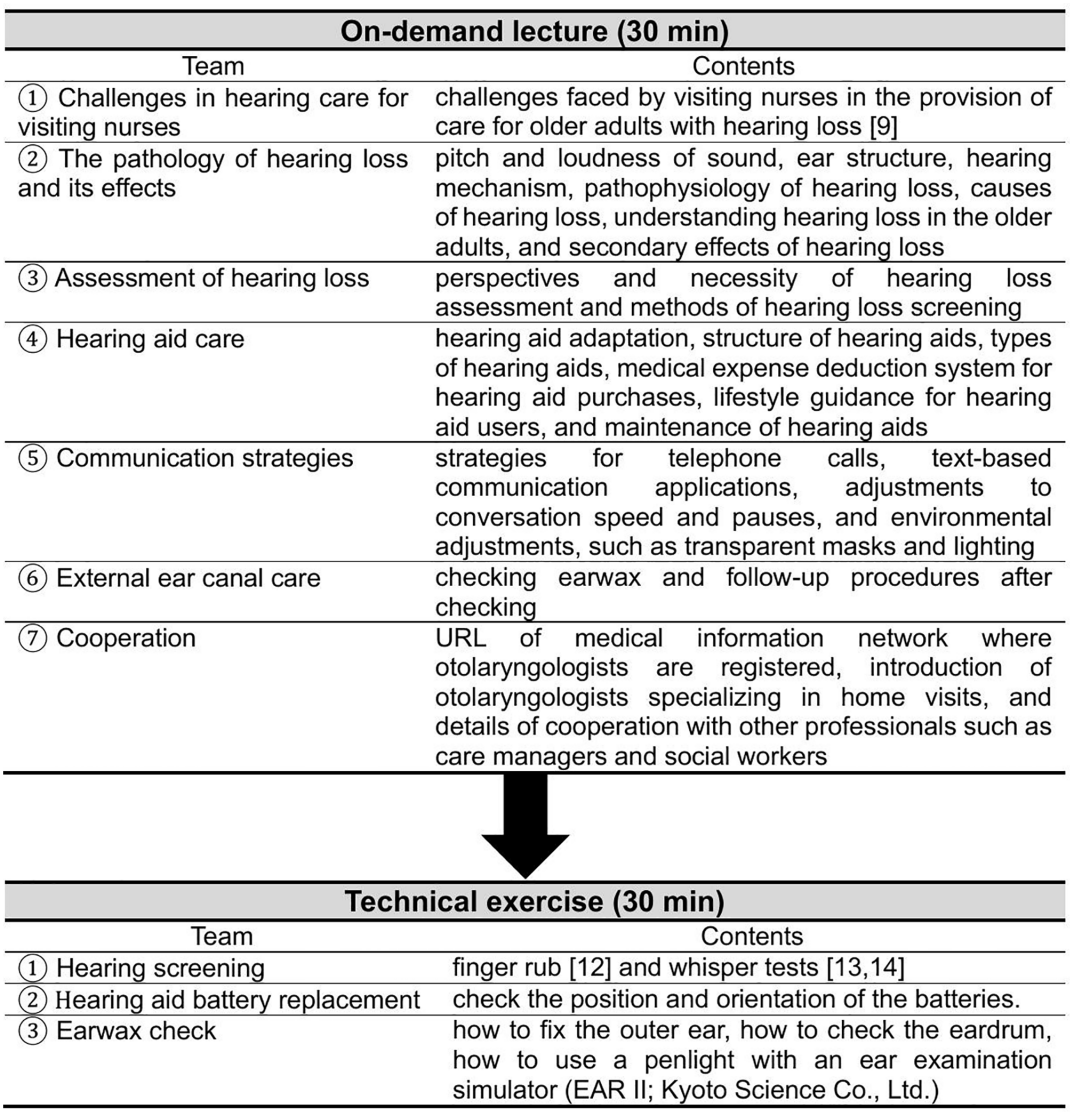
Hearing Loss Care Education Program for the Older Adults.

This program was an educational intervention designed to enable visiting nurses to learn specific assessment and care techniques for older adults with hearing loss. The program components were based on hearing care content identified as lacking by visiting nurses [8] and hearing care content that visiting nurses found challenging [9]. This program was developed under the guidance of specialists in otolaryngology, head and neck surgery, and certified hearing aid specialists to ensure the reliability and validity of the educational content. Fig 1 presents the “Hearing Loss Care Education Program for the Older Adults.” This program comprised an on-demand lecture (30 minutes), followed by a technical exercise (30 minutes). The on-demand lecture was structured as follows: ① challenges in hearing care for visiting nurses ② the pathology of hearing loss and its effects (pitch and loudness of sound, ear structure, hearing mechanism, pathophysiology of hearing loss, causes of hearing loss, understanding hearing loss in older adults, and secondary effects of hearing loss), ③ assessment of hearing loss (perspectives and necessity of hearing loss assessment and methods of hearing loss screening), ④ hearing aid care (hearing aid adaptation, structure of hearing aids, types of hearing aids, medical expense deduction system for hearing aid purchases, lifestyle guidance for hearing aid users, and maintenance of hearing aids), ⑤ communication strategies (strategies for telephone calls, text-based communication applications, adjustments to conversation speed and pauses, and environmental adjustments, such as transparent masks and lighting), ⑥ external ear canal care (checking earwax and follow-up procedures after checking), ⑦ and cooperation (URL of medical information network where otolaryngologists are registered, introduction of otolaryngologists specializing in home visits, and details of cooperation with other professionals such as care managers and social workers). The technical exercises consisted of hearing screening (finger rub [12] and whisper tests [13,14]), hearing aid battery replacement, and earwax checks. Before starting the technical training, the “Hearing Care Technical Checklist,” “behind-the-ear hearing aids,” “hearing aid batteries,” and “penlights” were distributed to the participants. Training was conducted according to the “Hearing Care Technical Checklist.” For hearing screening, a noise meter (NL-27, Rion Co., Ltd.) was used to practice hearing finger rubbing sounds and whispering at approximately 40 dB while confirming the sound level. For hearing-aid battery replacement, behind-the-ear hearing aids and hearing-aid batteries were used to insert the batteries into the correct positions and confirm their orientations. The earwax was examined using an ear examination simulator (EAR II; Kyoto Science Co., Ltd.). The participants practiced until they could confirm the presence of earwax in the simulator by correctly identifying the method of fixing the outer ear, direction of the penlight for checking the eardrum, and other details. Finally, the participants answered questions about the content of the “Hearing Care Education Program for Older Adults” and shared examples of how the knowledge and care skills acquired through the program could be applied to their daily work, concluding the session.

### Control group

The control group received normal visiting nursing care for the seven months of data collection.

### Measures

Recruitment began on December 15,2023 and ended on February 13, 2024, when the target number of participating facilities was reached. Data were collected between February and September, 2024. The intervention group completed a web questionnaire at baseline (T1), immediately after the intervention (T2), 1 month after the intervention (T3), and 3 months after the intervention (T4). The non-intervention and intervention groups completed a web questionnaire simultaneously.

### Participant characteristics

The survey investigated the following factors: age, sex, family living together, oldest member of the household, years of nursing experience, years of visiting nurse experience, permanent employment, academic background, qualifications, position, visiting older adults with hearing loss, facility affiliation with a speech therapist, work experience in otolaryngology, general training experience, and experience with hearing loss training.

### Primary outcome

The evaluation criteria for the hearing loss care educational program were based on the Kirkpatrick evaluation model [15]. “Level 2: Learning” measured the extent to which learners had acquired knowledge, skills, attitudes, and confidence. “Level 3: Behavior” measured the extent to which learners could apply what they have learned in the program on their job [15]. The primary outcome of this study was Level 3 Behavior of the Kirkpatrick Evaluation Model, focusing on the implementation of hearing care. The following eight items were surveyed: hearing screening, checking earwax, cooperation with otolaryngologists regarding earwax removal, communication techniques for older adults with hearing loss, sharing ideas with other professionals regarding effective communication with older adults with hearing loss, hearing aid care, coordination with hearing aid consultation doctors regarding hearing aid purchase and maintenance, and cooperation with certified hearing aid specialists regarding hearing aid purchase and maintenance. For the “Implementation of hearing loss care” item, a 6-point scale ranging from “1: Not implemented at all” to “6: Always implemented” was used for the survey.

### Secondary outcome

The secondary evaluation items of this study were based on Level 2: Learning of Kirkpatrick’s evaluation model and investigated “knowledge of hearing care” and “confidence in providing hearing care.” “Knowledge of hearing care” was assessed using a total of 20 items, including 3 items on “pathology of hearing loss;” 3, “hearing screening;” 7, “hearing aids;” 4, “communication strategies with older adults’ individuals with hearing loss;” 1, “external ear canal care procedures;” and, 2, “collaboration in hearing care.”

Knowledge was assessed using a three-point scale: “correct,” “incorrect,” and “unknown.” The scale was scored out of 20 points, with correct answers receiving 1 point and incorrect answers (including “don’t know”) receiving 0 points. “Confidence in implementing hearing care” was specifically related to technical training content and included 3 items on “hearing screening,” 2 on “hearing aid-related care,” and 1 on “earwax assessment,” totaling six items. The survey used a 6-point scale ranging from “1: Not at all (I do not think I can implement)” to “6: Very much so (I think I can implement).”

Primary and secondary outcome measures were developed based on the “Hearing Care Education Program for Older Adults,” and efforts were made to ensure content validity as much as possible under the guidance of certified otolaryngology head and neck surgery specialists and certified visiting nurses with expertise in home-visit nursing.

### Data analysis

All analyses were conducted in accordance with intention-to-treat (ITT) principles. The distribution of the subject attributes was confirmed using the Shapiro–Wilk test. Comparisons between the intervention and non-intervention groups at baseline were performed using t-tests, Mann–Whitney U tests, and chi-square tests. Fisher exact probability test was performed when cells with an expected frequency of < 5 accounted for 20% or more of all the cells. To clarify the intervention effect, primary and secondary outcomes were analyzed using generalized estimating equations with data from T1 to T4. This study was a cluster randomized controlled trial, and the consider the intracluster correlations were considered in the measurements of each outcome [16]. Generalized estimating equations are highly flexible analytical methods that can adjust for intracluster correlations and temporal changes, present the results as estimates, and include missing data in the analysis [16]. In generalized estimating equation models, when the response variable is ordinal, the probability distribution is set to the ordinal logit, and the link function is set to the cumulative logit. When the response variable was continuous, the probability distribution was set as linear, and the link function was set identically. The working correlation matrix structure was set to AR (1). If estimates could not be calculated, they were considered unstructured. Confounders were set as predictor variables and covariates to adjust for confounding.

All statistical analyses were performed using SPSS 28 (IBM Corp, Armonk, NY, USA), and results with P-values < 0.05 were deemed statistically significant.

## Results

### Participant characteristics

**Fig 2:**
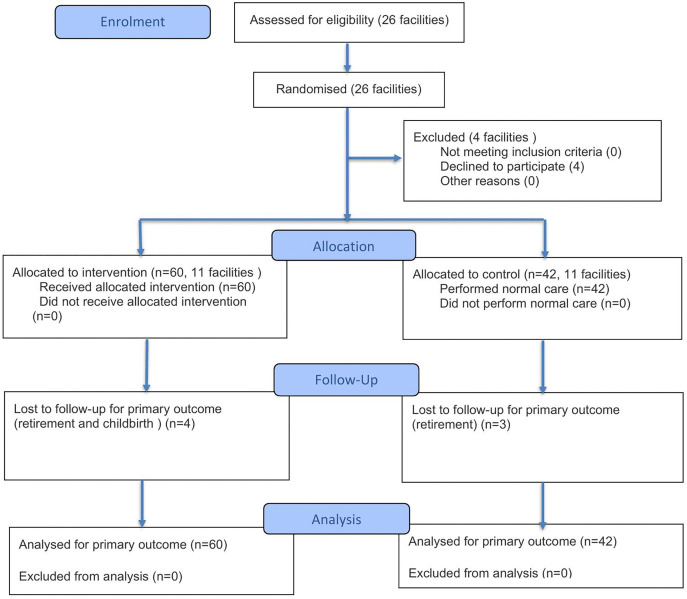
CONSORT 2025 Flow Diagram.

Fig 2 presents the CONSORT flowchart of the study participants. Of the 26 facilities that obtained permission to participate in the study, two facilities assigned to the intervention group and two facilities assigned to the control group withdrew prior to the commencement of the study (management and operational constraints). A total of 102 participants—60 visiting nurses from 11 facilities in the intervention group and 42 visiting nurses from 11 facilities in the non-intervention group—agreed to participate. Subsequently, 4 participants in the intervention group and 3 in the non-intervention group dropped out because of retirement and maternity leave, leaving 95 participants (56 and 39 in the intervention and non-intervention groups, respectively) who completed the follow-up survey. Following the ITT principle, the analysis included 60 and 42 participants in the intervention and non-intervention groups, respectively, with a total of 102 participants.

Table 1 presents the baseline characteristics of the participants. The only significant difference between the intervention and non-intervention groups at baseline was in the “facility affiliation of speech-therapist” (intervention group: n = 34 [56.7%], non-intervention group: n = 9 [21.4%], p < 0.001).

**Table 1:** Baseline characteristics of visiting nurses.

Variable	Intervention n = 60	Control n = 42	*p* ^1^
	n (%) or median (minimum value-maximum value)		
Age (year)	36.5 (30.0–43.8)	38.0 (31.0–48.0)	0.322
Sex			
Male	5 (8.3)	8 (19.0)	0.110
Female	55 (91.7)	34 (81.0)	
Family living together	42 (70.0)	34 (81.0)	0.212
Oldest member of the household	39.0 (30.5–48.5)	42.0 (31.8–48.3)	0.647
Years of nursing experience	11.0 (7.0–17.0)	12.5 (9.8–23.3)	0.193
Years of visiting nurse experience	2.0 (1.0–4.8)	2.5 (1.0–9.3)	0.388
Permanent employment	49 (81.7)	34 (81.0)	0.927
Academic background			
Professional training college	37 (61.7)	31 (73.8)	0.428
Junior college	5 (8.3)	1 (2.4)	
College	16 (26.7)	8 (19.0)	
Graduate school	2 (3.3)	2 (4.8)	
Qualification (multiple answers)			
Nurse license	58 (96.7)	42 (100.0)	0.511
Assistant nurse license	7 (11.7)	6 (14.3)	0.696
Public health nurse license	12 (20.0)	6 (14.3)	0.456
Care manager	0 (0.0)	2 (4.8)	0.167
Certified nurse	0 (0.0)	1 (2.4)	0.412
Certified nurse specialist	1 (1.7)	0 (0.0)	1.000
Position			
Staff	47 (78.3)	27 (64.3)	0.122
Director	1 (1.7)	4 (9.5)	
Administrator	12 (20.0)	11 (26.2)	
Visiting older adults with hearing loss			
Yes	57 (95.0)	39 (92.9)	0.688
No	3 (5.0)	3 (7.1)	
Facility affiliation of speech-therapist			
Yes	34 (56.7)	9 (21.4)	<0.001
No	26 (43.3)	33 (78.6)	
Work experience in otolaryngology			
Yes	13 (21.7)	4 (9.5)	0.176
No	47 (78.3)	38 (90.5)	
General training experience			
Yes	20 (33.3)	16 (38.1)	0.620
No	40 (66.7)	26 (61.9)	
Experience with hearing loss training			
Yes	1 (1.7)	5 (11.9)	0.079
No	59 (98.3)	37 (88.1)	

^1^To detect between-group differences, an independent sample Mann–Whitney U test was performed for continuous variables, and Fisher exact test was performed for categorical variables.

Table 2 shows the primary and secondary outcome measures at baseline. The only significant difference between the intervention and non-intervention groups at baseline was in “knowledge of hearing care” (median 7, interquartile range 5–10 in the intervention group; median 5.5, interquartile range 4–8 in the non-intervention group; p = 0.045).

**Table 2:** Baseline the primary and secondary outcome of visiting nurses.

Variable	Intervention n = 60	Control n = 42	
	Median (minimum value-maximum value)		*p* ^1^
**Primary outcome (implementation of hearing care)**			
Hearing screening	1 (1–2)	1 (1–2)	0.572
Checking earwax	3 (2–4)	3 (2–4)	0.604
Cooperation with otolaryngologists regarding earwax removal	1 (1–2)	1 (1–1.3)	0.295
Communication techniques for older adults with hearing loss	4 (4–5)	5 (4–6)	0.177
Sharing ideas with other professions on effective communication with older adults with hearing loss	3 (2–4)	4 (2–4)	0.202
Hearing aid care	2 (1–3)	2 (1–3)	0.454
Coordination with hearing aid consultation doctors regarding hearing aid purchase and maintenance	1 (1–1)	1 (1–1)	0.902
Cooperation with certified hearing aid specialists regarding hearing aid purchase and maintenance	1 (1–1)	1 (1–1)	0.903
**Secondary outcome (knowledge of hearing care, confidence in implementing hearing care)**			
Knowledge of hearing care	7 (5–10)	5.5 (4–8)	0.045
Finger rubbing sound test	3 (2–4)	3 (1–4)	0.770
Electronic thermometer alarm sound test	3 (2–4.8)	3 (1–4)	0.365
Whisper test	3 (1–4)	3 (1.8–4)	0.627
Explanation of how to prevent hearing aid howling	2 (1–3)	2 (1–3)	0.553
Replacing hearing aid batteries	3 (2–4.8)	4 (2–4.3)	0.871
Earwax assessment	3 (2–4)	4 (3–4)	0.227

^1^To detect between-group differences, an independent sample Mann–Whitney U test was performed for continuous variables.

### Primary outcome

Table 3 presents the results of the generalized estimating equations for the primary outcomes. The analysis was adjusted for confounding factors such as age, sex, speech-language pathologist at the facility, experience working in otolaryngology, and experience in hearing loss training. Among the 8 items related to “implementation of hearing care,” a significant between-group difference was observed in “implementation of hearing screening” (odds ratio 8.35, 95% confidence interval 3.60–19.34, p < 0.001).

**Table 3:** Between-group differences in primary outcomes.

Variable	odds ratio	95%CI	*P* ^1^
Hearing screening	8.35	3.60–19.34	<0.001
Checking earwax	1.64	0.84–3.20	0.146
Cooperation with otolaryngologists regarding earwax removal	2.02	0.90–4.52	0.088
Communication techniques for older adults with hearing loss	1.65	0.74–3.67	0.224
Sharing ideas with other professionals on effective communication with older adults with hearing loss	0.72	0.33–1.55	0.395
Hearing aid care	1.77	0.88–3.55	0.108
Coordination with hearing aid consultation doctors regarding hearing aid purchase and maintenance	1.36	0.54–3.43	0.515
Cooperation with certified hearing aid specialists regarding hearing aid purchase and maintenance	1.41	0.57–3.48	0.453

^1^Generalized estimation equation adjusted for confounding factors

### Secondary outcome

Table 4 presents the results of the generalized estimating equations for the secondary outcomes. In the analysis, we adjusted for confounding factors such as age, sex, language therapist affiliation, experience working in otolaryngology, and training in hearing loss. “knowledge of hearing care” showed a significant between-group difference (partial regression coefficient 3.34, 95% confidence interval 2.27–4.41, p < 0.001). Additionally, all six items of “confidence in implementing hearing care” showed significant between-group differences.

**Table 4:** Between-group differences in secondary outcomes.

Variable	Partial regression coefficient	odds	95% CI	*P* ^1^
Knowledge of hearing care	3.34		2.27–4.41	<0.001
Finger rubbing sound test		5.99	3.14–11.41	<0.001
Electronic thermometer alarm sound test		5.93	3.18–11.08	<0.001
Whisper test		4.81	2.55–9.08	<0.001
Explanation of how to prevent hearing aid howling		5.48	2.77–10.82	<0.001
Replacing hearing aid batteries		4.28	2.22–8.23	<0.001
Earwax assessment		3.62	2.00–6.54	<0.001

^1^Generalized estimation equation adjusted for confounding factors.

## Discussion

### Participant characteristics

There were significant differences between the intervention and non-intervention group in terms of “speech-therapist employed at the facility” and “knowledge of hearing care.” The average number of rehabilitation professionals employed at home visit nursing stations in Japan is 1.44 physical therapists, 0.63 occupational therapists, and 0.18 speech therapists [17], with a particularly low number of speech therapists. Furthermore, to strengthen facility functions, the number of units provided by physical therapists and others was reduced from 297 units per visit to 293 units per visit [18], potentially limiting the ability of home-visit nursing stations with stable operation to employ rehabilitation professionals. Facilities were stratified based on the number of home-visit nurses at the time permission was obtained from facility managers and randomly assigned to the intervention and non-intervention groups. However, four facilities dropped out after allocation, and the number of visiting nurses who provided consent differed from that reported by the facility managers at the time of permission, resulting in a bias toward facilities with more visiting nurses in the intervention group (six facilities in the intervention group and three in the non-intervention group had five or more visiting nurses). These factors may have influenced the between-group differences among the speech therapists employed at the facility.” Additionally, the median score for “knowledge of hearing care” was 7 points in the intervention group and 5.5 points in the non-intervention group, with the intervention group showing a significantly higher score. Although the factors contributing to the intergroup differences in participant characteristics could not be identified, the intervention group had a significantly higher number of “facility-affiliated speech therapists.” In Japan, speech therapists have national qualifications with specialized knowledge of hearing and hearing loss, and they perform the roles of both “speech–language pathologists” and “audiologists.” Therefore, it can be considered that the intervention group, which had many “facility-affiliated speech therapists,” had more opportunities to learn about hearing care daily, leading to the significantly higher “knowledge of hearing care” in the intervention group.

### Effectiveness of the hearing loss care education program

This program aimed to improve the comprehensive hearing care capabilities of visiting nurses by providing knowledge about hearing care for assessment and implementation. The intervention had a significant effect on the primary outcome of “implementation of hearing care,” specifically in “implementation of hearing screening.” According to Smith et al. [19], 87% of healthcare professionals do not conduct hearing screenings, primarily because they do not know how to do so. This program explained the perspective and necessity of hearing loss assessment as well as the procedures for hearing loss screening. Subsequently, the participants practiced using a sound level meter to accurately screen for hearing loss by confirming the volume of finger rubbing and whispering sounds through technical exercises. As a result, visiting nurses are believed to have gained an understanding of the necessity and procedures for hearing loss screening and are now able to conduct screening with confidence. Previous studies on hearing screening have reported that the finger-rubbing sound test sensitivity is 91% and the specificity is 68% [20], whereas the whisper test sensitivity is 82.9% and the specificity is 94.3% [21]. Hearing screening that does not require special equipment and can be performed quickly is important for primary care for older adults [20]. For visiting nurses who face the challenge of providing hearing loss care within the time allocated for home visits [9], hearing loss screening, which can be performed quickly, may be a feasible option for hearing loss care. Furthermore, the intervention showed a significant effect on the secondary outcome, “knowledge of hearing care”. Facility nurses were provided lectures and exercises on ear anatomy, risks of earwax blockage, and earwax removal methods, and it was reported that their understanding of this knowledge improved after the intervention [6]. This program was effective in improving knowledge acquisition, corroborating previous studies.

However, at baseline (T1), the intervention group already had significantly higher “knowledge of hearing care” than the non-intervention group (p = 0.045), which may have influenced the results of this study. All items of the secondary outcome, “confidence in implementing hearing care,” showed a significant effect of the intervention, indicating that the intervention group gained confidence in their ability to implement hearing care. This suggests that the program not only improved the knowledge and understanding of hearing care but also enabled participants to practice and master the necessary skills through technical exercises, which likely contributed to their increased confidence. The proportion of participants in this study with prior experience in hearing loss training was 2–12%, which was lower than the 36% reported in a previous study on hearing aid training [22] and approximately 60% reported in this study for “general training experience.” Given that the participants had not received education on hearing care, the fact that they were able to gain confidence in implementing hearing care despite their anxiety as visiting nurses suggests that the program’s content was highly effective. However, there was no significant effect on “hearing aid care.”. When providing hearing aid care to older adults with hearing loss, 78% of healthcare professionals reported needing information on hearing aid maintenance [22], and over 90% of visiting nurses in Japan reported needing training in hearing aids [8]. However, as hearing aid care is only necessary when older adults with hearing loss use hearing aids, the lack of opportunities for hearing aid care may have influenced the results. There were no differences in earwax care between groups, including earwax checks and coordination with otolaryngologists for earwax removal. Due to difficulties in preparing the necessary items for ear cleaning at home [9], the program instructed visiting nurses to use a penlight that they always carried to check for earwax. However, visiting nurses found it challenging to perform ear cleaning within the visit time [9], and “earwax care” may have had lower priority compared to other care tasks within the limited time, potentially reducing the effectiveness of the intervention.

### Strengths and limitations

The strengths of this study are as follows: (1) the program was developed based on the actual practices and challenges of visiting nurses in providing hearing care and was guided by specialists in otolaryngology-head and neck surgery and certified hearing aid specialists to ensure program quality; (2) the validity of the study results was ensured through a cluster randomized controlled trial; and, (3) the influence of intra-cluster correlation was eliminated, enabling the evaluation of longitudinal data. However, this study had several limitations: (1) given that participants were recruited only in Aichi Prefecture, there may have been selection bias in their characteristic; (2) because dice were used for randomization, reproducibility may not be guaranteed; (3) in the randomization process, participants who dropped out of the study after being assigned to either the intervention or control group may have affected the allocation; (4) participants were verbally informed that the effectiveness of the intervention was unknown. However, blinding after randomization was difficult, and the intervention group may have had expectations regarding the acquisition of hearing care skills; (5) there were no measures to evaluate hearing care, so the reliability and validity of the outcomes were limited because the primary and secondary outcomes were created by us; (6) the study period was limited, and the primary outcome was the implementation of hearing loss care by visiting nurses. It was not possible to evaluate the outcomes of older adults who received; and, (7) The study population comprised visiting Japanese nurses. Therefore, these findings may not be generalizable to non-Japanese populations.

### Implications for future research

Previous research on hearing care education for healthcare professionals has primarily focused on facility-based nurses and non-healthcare professionals in community settings [6,23]. To date, no hearing care education program has been developed specifically for visiting nurses. This study aimed to improve the comprehensive hearing loss care skills of home care nurses, from knowledge acquisition to assessment and implementation. These findings provide important insight into how visiting nurses can develop and apply hearing loss care skills in practice. Future research should extend the study period to evaluate the effectiveness of the program and its impact on the outcomes of older adults with hearing loss. Furthermore, although this study conducted technical exercises of the hearing-impairment care education program in a face-to-face format, future initiatives should ensure feasibility while considering the use of applications and other tools within the program.

## Conclusions

Our findings indicate that the “Hearing Loss Care Education Program for older adults” helped participants acquire knowledge about hearing care, increased their confidence in providing it, and enabled them to undergo hearing screening.

## Supporting information

S1 Table CONSORT 2025 checklist. (DOCX)

S2 Table Web survey data. (XLSX)

S1 Fig Temporal changes in primary outcome. (TIF)

S2 Fig Temporal changes in secondary outcome. (TIF)
